# Genome-Wide Location Analysis Reveals Distinct Transcriptional Circuitry by Paralogous Regulators Foxa1 and Foxa2

**DOI:** 10.1371/journal.pgen.1002770

**Published:** 2012-06-21

**Authors:** Irina M. Bochkis, Jonathan Schug, Diana Z. Ye, Svitlana Kurinna, Sabrina A. Stratton, Michelle C. Barton, Klaus H. Kaestner

**Affiliations:** 1 Department of Genetics and Institute for Diabetes, Obesity, and Metabolism, University of Pennsylvania School of Medicine, Philadelphia, Pennsylvania, United States of America; 2 Center for Stem Cell and Developmental Biology, Department of Biochemistry and Molecular Biology, University of Texas M. D. Anderson Cancer Center, Houston, Texas, United States of America; Stanford University School of Medicine, United States of America

## Abstract

Gene duplication is a powerful driver of evolution. Newly duplicated genes acquire new roles that are relevant to fitness, or they will be lost over time. A potential path to functional relevance is mutation of the coding sequence leading to the acquisition of novel biochemical properties, as analyzed here for the highly homologous paralogs Foxa1 and Foxa2 transcriptional regulators. We determine by genome-wide location analysis (ChIP-Seq) that, although Foxa1 and Foxa2 share a large fraction of binding sites in the liver, each protein also occupies distinct regulatory elements *in vivo*. Foxa1-only sites are enriched for p53 binding sites and are frequently found near genes important to cell cycle regulation, while Foxa2-restricted sites show only a limited match to the *forkhead* consensus and are found in genes involved in steroid and lipid metabolism. Thus, Foxa1 and Foxa2, while redundant during development, have evolved divergent roles in the adult liver, ensuring the maintenance of both genes during evolution.

## Author Summary

The duplication of a gene from a common ancestor, resulting in two copies known as paralogs, plays an important role in evolution. Newly duplicated genes must acquire new functions in order to remain relevant, otherwise they are lost via mutation over time. We have performed genome-wide location analysis (ChIP–Seq) in adult liver to examine the differences between two paralogous DNA binding proteins, Foxa1 and Foxa2. While Foxa1 and Foxa2 bind a number of common genomic locations, each protein also localizes to distinct regulatory regions. Sites specific for Foxa1 also contain a DNA motif bound by tumor suppressor p53 and are found near genes important to cell cycle regulation, while Foxa2-only sites are found near genes essential to steroid and lipid metabolism. Hence, Foxa1 and Foxa2 have developed unique functions in adult liver, contributing to the maintenance of both genes during evolution.

## Introduction

Expansion of transcription factor gene families has greatly contributed to the complexity of metazoan genomes [1]. Newly duplicated genes must acquire new functions in order to remain relevant, otherwise they are lost via mutation over time. Transcription factor paralogs diversify either by a mutation arising in *cis*-regulatory elements, leading to novel expression patterns, or by divergence in their coding sequence, acquiring new functions [2]. A modification of the DNA binding domain can alter the consensus sequence of a transcription factor, while a change in the interacting domain allows the protein to interact with new partners and thereby gain new gene targets.

Functional diversification of DNA-binding proteins is of particular importance since transcription factors control regulatory networks that both direct cell specification and patterning in development and govern cellular homeostasis in differentiated tissues. While their main function during development is in directing correct pattern formation [3], transcription factors in the adult organisms need to be adaptive to different physiological conditions and respond to variety of signals in the same cell [4]. Hence embryonic gene regulatory networks are “overwired”, having multiple subnetworks with redundant functions where transcription factors are used numerous times in different modules to specify structures in different spatiotemporal context, while networks in differentiated tissues are designed to be more flexible.

The developmental regulators Foxa1 and Foxa2, members of the winged helix transcription factor family, share a highly conserved 100 amino acid DNA binding domain, and have been shown to cooperate and direct early liver and pancreas development [5]. In addition, Foxa2 plays an important role in bile acid metabolism in the adult liver [6]. Foxa2 is required to prevent intrahepatic cholestasis and liver injury in mice fed a cholic acid-enriched diet. Furthermore, expression of FOXA2 is markedly decreased in liver samples from individuals with different cholestatic syndromes. Hence, genetic evidence suggests that Foxa1, the closest paralog to Foxa2, cannot fully compensate for the loss of Foxa2 in the liver.

Here we investigated whether alterations in the biochemical properties of close paralogs, as shown by genome-wide target preferences *in vivo*, contribute to their evolutionary diversification. We find that Foxa1 and Foxa2, while redundant during development, are functionally diversified in the adult liver through target occupancy, ensuring their evolutionary fitness as distinct regulators of transcription.

## Results

### Foxa1 and Foxa2 cooperate to regulate gene expression in the fetal liver, but diverge to direct transcription in the adult liver

**Figure 1:**
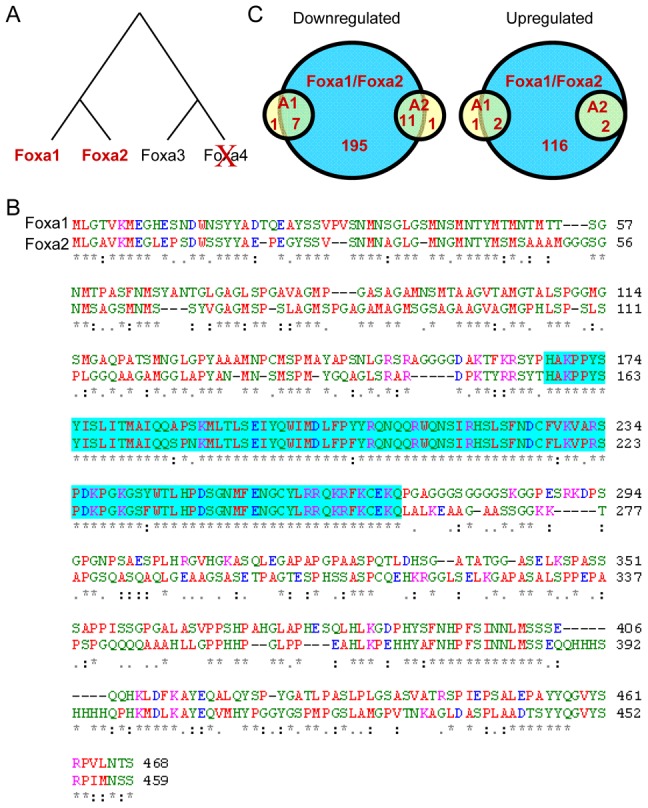
Evolution of Vertebrate Foxa Paralogs and Functional Redundancy in the Fetal Liver. (A) Phylogenetic tree of the Foxa subfamily of transcriptional regulators. The putative Foxa4 gene was lost in vertebrates during evolution. (B) Sequence alignment of mouse Foxa1 and Foxa2 proteins by ClustalW2 algorithm. The winged-helix DNA binding domain is highlighted in blue. ‘*’, identical residues in all sequences, ‘:’ highly conserved amino acids; ‘.’ weakly conserved amino acids. (C) Venn diagram of the number of genes that are differentially expressed in fetal livers of Foxa1 and Foxa2 single mutants, as well as the double mutant (shaded in blue) on embryonic day 18.5. The number of genes dependent on each single factor is small compared to the number of genes that are differentially expressed in the double mutant.

Gene duplication followed by functional diversification of the duplicates is a primary driver of evolution. The developmental regulators Foxa1 and Foxa2, the closest paralogs in the Foxa subfamily of winged-helix transcription factors (Figure 1A), have been shown to collaborate to initiate liver development [5], [7]. The two proteins share a highly similar 100 amino acid DNA binding domain [8], [9], and strong sequence conservation in their transactivation domains, located at both N and C termini [10], [11] (Figure 1B). However, structural differences exist as well: threonine 156 of Foxa2 has been suggested as an Akt phosphorylation site [12], and serine 283 has been proposed as a phosphoacceptor for DNA-dependent protein kinase [13].

In order to ascertain the relative regulatory contributions of Foxa1 versus Foxa2 during late fetal hepatic development, we assembled gene expression profiles of mouse liver tissue of Foxa1 and Foxa2 individual mutants, as well as double mutants (*Foxa1* ^−/−^;*Foxa2^loxP/loxP^*;*Alfp.Cre*), by analyzing RNA isolated on embryonic day 18. The number of genes dependent on each single factor was quite limited, compared to the number of genes that were differentially expressed in the double mutant (Figure 1C), suggesting that Foxa1 and Foxa2 remain largely redundant at this late-fetal stage of liver development. This functional redundancy is supported by the fact that when both genes are ablated using the Cre/loxP system during fetal liver development in *Foxa1^loxP/loxP^Foxa2^loxP/loxP^AlfpCre* mice, biliary hyperplasia ensues, which is not seen when either gene is conditionally ablated by itself [14].

**Figure 2:**
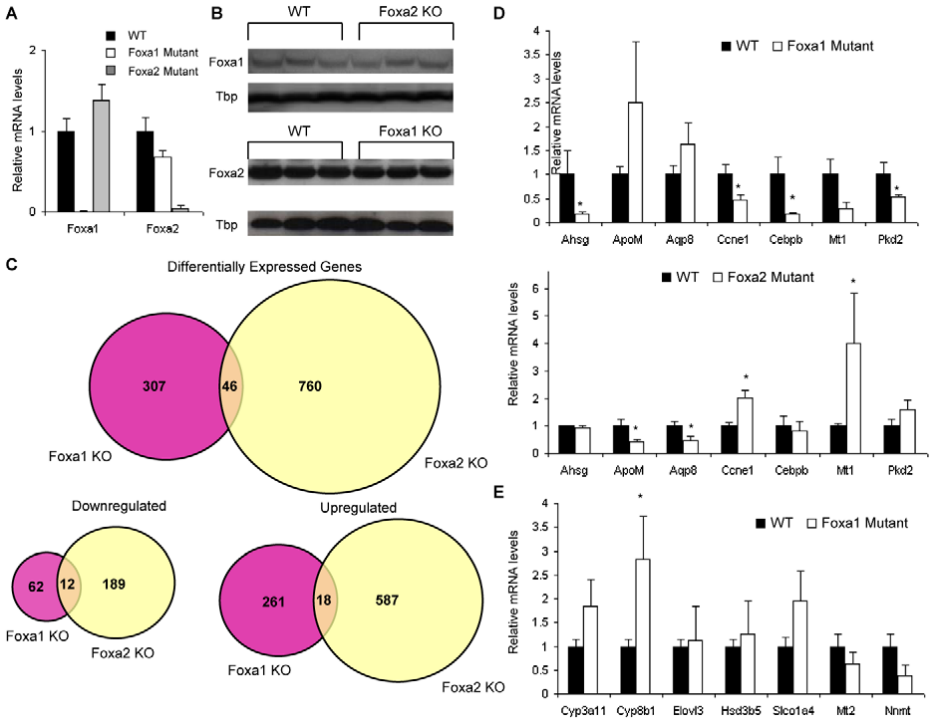
Foxa1 and Foxa2 Regulate Different Sets of Target Genes in the Adult Liver. (A,B) Expression of Foxa factors in reciprocal mutant mice. Black bar, wild-type liver, white bar, Foxa1-deficient liver, and grey bar, Foxa2-deficient liver. As expected, Foxa1 mRNA is undetectable in the Foxa1 Foxa1-deficient liver, while expression of Foxa2 is not changed in absence of Foxa1 compared to control mice on both the mRNA and protein level. Similarly, expression of Foxa2 is near background levels in Foxa2-deficient livers, while expression of Foxa1 is comparable to that in wild-type littermates. (C) Venn diagrams of the number of genes that are differentially expressed (|FC|> = 1.5 and FDR = 15%) in adult livers (total, downregulated, and upregulated) of Foxa1 (pink circles) and Foxa2 mutant (yellow circles) mice. (D) Confirmation of seven microarray targets changed in expression in the Foxa1-deficient liver by quantitative real-time PCR (qRT-PCR) (top panel). When the mRNA levels of the same genes were determined in the Foxa2-deficient liver (bottom panel), these targets were either not Foxa2-dependent at all or regulated in the opposite direction. Values are represented as means plus standard error. P values were determined by Student's *t* test. * p-value<0.05. (E) mRNA levels of previously identified Foxa2 targets in Foxa1 mutant mice. Values are represented as means plus standard error.

Next, we assessed the involvement of Foxa1 and Foxa2 in the control of the transcriptional program in the adult liver. One concern regarding the analysis of Foxa1- or Foxa2-specific binding and regulation of gene expression is the possibility of mutual compensation. In other words, if one of the factors is missing, the other might be increased in expression, occupy the previously unique binding sites, and affect expression of previously unique targets. To address this issue, we determined the expression of Foxa factors in the livers of reciprocally mutated mice. In Foxa1-deficient livers, expression of Foxa1 is virtually undetectable, while expression of Foxa2 is not changed as compared to wild-type littermates at both mRNA and protein levels (Figure 2A, 2B). Foxa2 expression is lost in Foxa2 mutant mice as expected, while expression of Foxa1 is comparable to that of control mice. Hence, for both Foxa1 and Foxa2, when one factor is deleted, the paralog does not compensate for that factor by a change in expression.

We determined gene expression profiles for adult mouse liver from Foxa1 and Foxa2 [4] single mutants and observed that overlap among the differentially-expressed genes between the two mutants is limited (Figure 2C). We verified mRNA levels of several microarray targets by quantitative real-time PCR (Q-PCR, Figure 2D) and determined that when expression of a certain target is significantly altered in one mutant, mRNA levels of this target are either not changed or follow the opposite pattern in livers of reciprocal mutant mice. This observation also holds true for previously published Foxa2 targets [4], which are regulated differently by Foxa1 (Figure 2E). Thus, in the adult liver, Foxa1 and Foxa2 do not fully compensate for each other, in contrast to the situation in the fetal organ.

### Identification of genomic targets of Foxa1 and Foxa2 in adult liver reveals common and factor-specific binding

**Figure 3:**
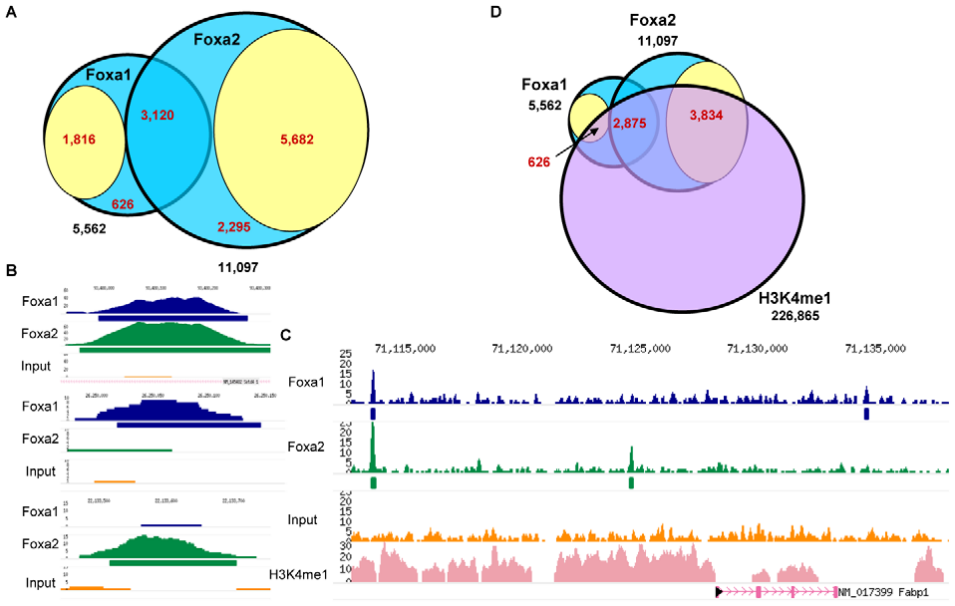
Identification of Genomic Targets of Foxa1 and Foxa2 in Adult Liver. (A) Venn diagram showing the results of genome-wide location analysis for Foxa1 and Foxa2 in the adult liver, identifying 5,562 binding sites for Foxa1 and 11,097 for Foxa2, of which 3,120 were called bound by both factors by the GLITR algorithm (certain common targets). Many of the apparently Foxa1- or Foxa2-specific regions contained overlapping reads from the opposite factor, indicating that the sites may be common, but the reads did not reach significant depth to be called by GLITR. We defined more stringent criteria for Foxa1-only and Foxa2-only binding sites. The sets of unique targets (yellow circles) contain peaks for the first factor that include at most one tag per million per KB for the other factor (1,816 Foxa1 sites with one or no Foxa2 tag and 5,682 Foxa2 sites with one or no Foxa1 tag). (B) Foxa1 and Foxa2 can occupy common binding sites (top panel) in the adult liver, or sites specific to either factor (Foxa1-only binding site, middle panel; Foxa2-only binding site, bottom panel). (C) Both common and unique binding sites for Foxa1 and Foxa2 can co-occur at a single genomic locus. (D) Comparison of Foxa1 and Foxa2 binding sites to a profile of H3K3me1 regions (purple circle) in the adult mouse liver.

Next, in order to examine how DNA binding influences differential gene regulation in the adult liver, we analyzed data from genome-wide location analysis (ChIP-Seq) for Foxa1 and Foxa2. We identified 5,682 binding sites for Foxa1 and 11,097 for Foxa2 using the GLITR algorithm [15], of which only 3,120 sites were bound by both factors (FDR 5%, Figure 3A). However, in many of the apparently Foxa1- or Foxa2-specific regions there were overlapping sequence reads from the opposite factor, indicating that the sites may potentially be common to both proteins but that the sequence reads did not reach sufficient depth. To identify truly unique binding sites for each factor for subsequent analyses, we defined stringent criteria for Foxa1-only and Foxa2-only binding sites. The set of unique targets (Figure 3A, yellow circles) contains peaks for the first factor that include at most one tag per million per kilobase (Kb) for the other (1,816 Foxa1 sites with one or no Foxa2 tag and 5,682 Foxa2 sites with one or no Foxa1 tag). Specific examples of both common and unique Foxa1/Foxa2 targets are shown in Figure 3. Foxa1 and Foxa2 can occupy common binding sites (Figure 3B, top panel) in the adult liver, or sites specific to either factor (Foxa1 binding site, Figure 3B, middle panel vs. Foxa2 binding site, Figure 3B, bottom panel). An intriguing case is shown in Figure 3C, where both common and unique binding events co-occur at a single locus.

Chromatin-association of both Foxa1 and Foxa2 was previously correlated with the methylation status of histone H3 lysine 4 [16], [17]. Mono-methylated H3K4 (H3K4me1) is enriched at distal enhancers; therefore, we compared Foxa1 and Foxa2 binding sites to a profile of H3K3me1 regions in the adult murine liver [17]. While 92 percent of common Foxa1/Foxa2 targets and a majority of Foxa2-unique sites (68%) were found in H3K4me1 domains, surprisingly, only twenty-nine percent of Foxa1-specific regions co-localized with H3K4me1 blocks (Figure 3D), indicating another functional difference between Foxa1 and Foxa2 in the adult liver. An example is shown in Figure 3C, where both common and Foxa2-specific binding events are located in H3K4me1 domains, while a Foxa1-unique site occurs in the H3K4me1-free region.

**Figure 4:**
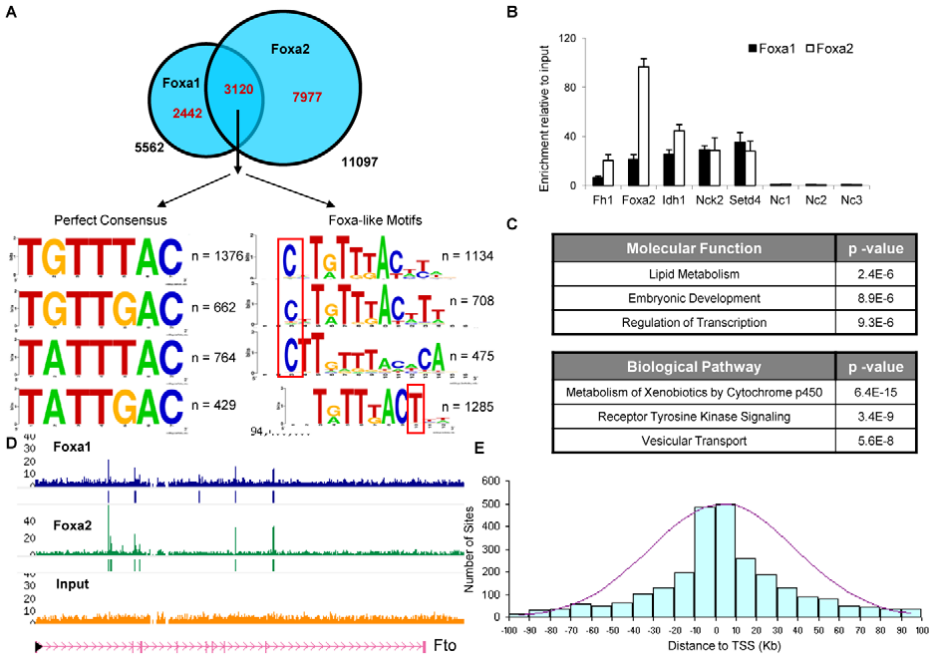
Dual Foxa1/Foxa2 Sites Contain a Perfect *Forkhead* Motif. (A) All sequences bound by both Foxa1 and Foxa2 in the liver contain at least one perfect match to the Foxa consensus (a 7-mer of 4 possible sequences, with variation in the second and fifth nucleotide, (T[A/G]TT[G/T]AC)), and often an additional Foxa-like motif, containing one or two degenerate nucleotides. Surprisingly, these sites show additional preferences for specific bases at positions flanking the *forkhead* motif, with a “C” occurring frequently one nucleotide upstream, and a “T” enriched immediately downstream of the core consensus sequence. (B) Confirmation of several common Foxa1/Foxa2 targets by quantitative RT-PCR and three negative control regions (with low amount of reads, Nc1, Nc2, and Nc3). Binding is expressed as enrichment of immunoprecipitated DNA relative to input DNA in liver chromatin. (C) Functional categories over-represented in the genes bound by both Foxa1 and Foxa2 include ‘embryonic development’, ‘regulation of transcription’, and ‘lipid metabolism’. Biological pathways enriched for Foxa1/Foxa2 targets are ‘metabolism of xenobiotics’, ‘vesicular transport’ and ‘receptor tyrosine kinase signaling’. (D) Foxa1 and Foxa2 bind to four distinct intronic regions of *Fto* (fat mass and obesity associated) gene, which is associated with the risk of diabetes. (E) Histogram of *cis*-regulatory elements bound by both Foxa paralogs shows a normal distribution near transcription start sites (TSS), with most sites within ten kilobases (Kb) from TSS.

Next, we analyzed DNA sequences present in common and unique binding sites in detail using a variety of computational tools. Sites that are bound by both Foxa1 and Foxa2 possess unique properties. Performing *de novo* motif analysis of these sequences, we found that all target sequences contain at least one perfect match to the previously known Foxa consensus binding site (a 7-mer of 4 possible sequences, with variation in the second and fifth nucleotide, (T[A/G]TT[G/T]AC), and frequently also a second Foxa-like motif, containing one or two degenerate nucleotides (Figure 4A). In addition, we scanned the sequences with established positional weight matrices (PWMs) from the Jaspar [18] and Transfac databases [19]. The top motifs enriched and identified as statistically significant among the common targets were numerous *forkhead* motifs, with the lowest p-value corresponding to the matrix for FOXD1 (FOXD1/Jaspar, FREAC4/Transfac, Table S1). Thus, both *de novo* motif finding and PWM scan analysis demonstrated that all sites bound by both Foxa paralogs contain a strong *forkhead* consensus sequence. Strikingly, we observed that sites common to Foxa1 and Foxa2 also have preferences for sequences flanking the *forkhead* motif, a “C” one nucleotide upstream and a “T” immediately downstream of the core consensus (Figure 4A). Hence, in addition to the well-known consensus, other nucleotides are likely important for binding by Foxa factors.

We validated several dual Foxa1 and Foxa2 targets by quantitative real-time PCR analysis of ChIP-enriched DNA (Figure 4B). Functional categories over-represented among the genes bound by both Foxa1 and Foxa2 included ‘embryonic development’, ‘regulation of transcription’, and ‘lipid metabolism’ (Figure 4C). These results are consistent with previous reports showing these factors are necessary for initiation of liver development [5] and that Foxa2 is required for normal bile acid homeostasis in the adult liver [6]. ‘Metabolism of xenobiotics by cytochrome p450’, important for hepatocyte function, and ‘vesicular transport and secretion’, previously associated with Foxa proteins in the endocrine pancreas [20], are among biological pathways enriched as well. Interestingly, Foxa1 and Foxa2 also occupy *cis*-regulatory elements of many diabetes susceptibility genes, both those mutated in MODY (mature onset diabetes of the young) and those with alleles associated with diabetes risk identified by genome-wide association studies (GWAS) (Table S2). An example is the genomic locus of *Fto* (fat mass and obesity associated gene), where Foxa1 and Foxa2 bind four distinct intronic regions (Figure 4D). These findings suggest that variant alleles of *FOXA1* and *FOXA2* might also contribute to the diabetes risk in human populations.

Among the Foxa1/Foxa2 targets are twelve nuclear hormone receptors and multiple liver-enriched DNA binding proteins (Hnf1a, Hnf1b, Hnf4a, Hnf6, Onecut2, Cebpa, Gata6, Hes1, Hhex, Prox1). In addition, Foxa1 and Foxa2 bind regulatory regions of three members of the CTF/nuclear factor I family (Nfia, Nfib, and Nfix) and the transcriptional repressor CTCF. Interestingly, the recognition motifs for Hnf1a, Hnf4a, Hnf6, and the nuclear factor I family members are also enriched in the target sequences bound by both Foxa paralogs (Table S3), thus suggesting distinct feed-forward regulatory loops in the network of genes bound by Foxa1 and Foxa2. Dual Foxa1/Foxa2 sites are distributed near transcription start sites (TSS), with most sites within ten kilobases (Kb) from TSS (Figure 4E).

### Foxa1-only sites have a weak forkhead consensus, are bound by p53, and are associated with cell cycle genes

**Figure 5:**
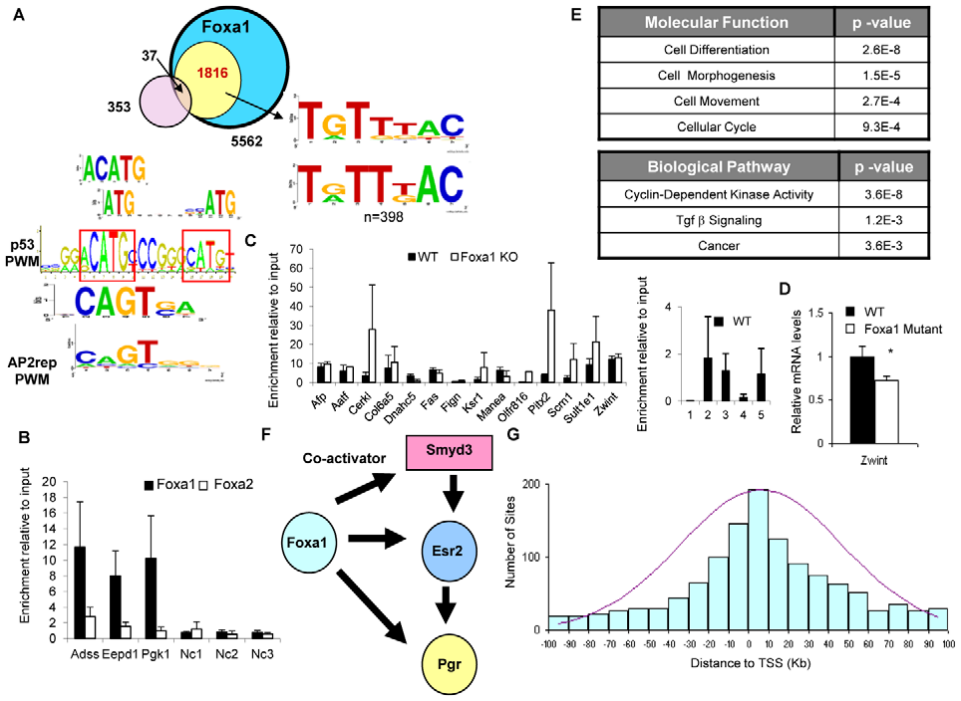
Foxa1-Specific Targets Are Enriched for p53 Binding Sites. (A) A set of Foxa1-only targets was defined as those sequences that had one or fewer sequence tags Foxa2 ChIP-Seq data set (yellow circle). Motif analysis of these sequences found in a weaker *forkhead* consensus. In addition to the *forkhead* consensus, several other motifs appeared in this set of sequences. The first (ACATG and ATG repeats with a spacer in the middle) comprises parts of the positional weight matrix (PWM) for p53. The second closely resembles the PWM of Klf12, also known as repressor of AP-2alpha (Tfap2a). Orthogonal analysis of sites bound only bound by Foxa1 (yellow circle) and gene expression changes in livers of Foxa1-deficient mice (light purple circle) shows that thirty-seven are direct targets of Foxa1. (B) Verification of Foxa1-only targets by qPCR. Filled bars, ChIP of liver chromatin with an anti-Foxa1 antibody, open bars, ChIP with an anti-Foxa2 antibody. Three negative control regions (Nc1, Nc2, and Nc3) with a low amount of reads are included Binding is expressed as enrichment of the PCR amplicon relative to input DNA in liver chromatin. (C) Binding of p53 to *cis*-regulatory elements of its previously identified targets (positive controls), the alpha-fetoprotein (AFP) and TNF receptor superfamily *Fas* genes and twelve additional Foxa1-only targets, including cell-cycle associated *Aatf* and *Zwint* genes in both wildtype (black bars) and Foxa1 mutant livers (white bars) (left panel) by ChIP of liver chromatin followed by qPCR. None of Foxa2-only sites tested were bound by p53 in wiltype livers (right panel) (D) Quantitative RT- PCR analysis for mRNA of Zwint, Zwint mRNA levels are significantly downregulated in livers of Foxa1 mutant mice by 30%, Values are represented as means plus standard error. P values were determined by Student's *t* test. * p-value<0.05 (E) Foxa1 targets are enriched in genes involved in cell differentiation, morphogenesis, movement, and cellular cycle. (F) A regulatory feed-forward loop involving Foxa1. In the set of Foxa1-specific targets, Foxa1 binds to regulatory elements of the nuclear receptor Esr2 (estrogen receptor), its co-activator Smyd3, and target gene Pgr (progesterone receptor). (G) Foxa1 only sites are more distributed more broadly surrounding TSS (+/−100 Kb) than dual Foxa1/Foxa2 targets (compare to Figure 4E).

Next, we performed *de novo* motif analysis using the sequences bound by Foxa1 only, as defined above. The top motif in the Foxa1-only set (yellow circle) was a weak *forkhead* motif, with three-hundred ninety-eight (or twenty-two percent) sequences containing a perfect Foxa consensus (T[A/G]TT[G/T]AC) (Figure 5A). Results of scanning the sequences with positional weight matrices (PWMs) resulted in enrichment of 14 forkhead PWMs (Table S1).

In addition to the *forkhead* consenus, several other motifs appeared in the Foxa1-only set. The first (ACATG and ATG repeats with a spacer in the middle, Figure 5A) comprise portions of the positional matrix for p53, which is also enriched in both conservative and semi-conservative sets by scanning analysis. The second motif closely resembles the PWM of Klf12, also known as repressor of AP-2alpha (Tfap2a), a transcription factor that interacts with p53 [21].

Orthogonal analysis of sites bound only bound by Foxa1 (Figure 5A, yellow circle) and gene expression changes in livers of Foxa1-deficient mice (Figure 5A, light purple circle) shows that thirty-seven are direct targets of Foxa1. We have shown that changes in gene expression in Foxa2 mutants is dependent on physiological state examined [4]. For instance, the number of Foxa2 direct targets increases significantly on a cholic-acid enriched diet as compared to standard chow. Hurtado and colleagues have reported that FOXA1 is necessary for estrogen to regulate expression of numerous genes in breast cancer cells [22], and we have just shown that Foxa1 and Foxa2 cooperate in mediating the effects of estrogens and androgens on liver cancer risk in response to carcinogens [23]. While deletion of Foxa1 does not change expression of many genes in the basal state in quiescent liver, it is thus likely that Foxa1 regulates mRNA levels of numerous targets in other physiological conditions, such as proliferation, estrogen response, or androgen response. We also verified that several ChIP targets in this group were indeed bound only by Foxa1 by Q-PCR (Figure 5B).

To investigate whether the presence of the motif resembling the p53 PWM among Foxa1-only targets truly indicated binding by p53, we performed chromatin immunoprecipitation analysis in livers of adult wild type mice. p53 occupied the regulatory regions of previously identified targets, including the alpha-fetoprotein (AFP) and TNF receptor superfamily *Fas* genes (Figure 5C, left panel). To confirm our predictions from the genome-wide location analysis for Foxa1, we examined p53 binding in twelve additional *cis*-regulatory elements of Foxa1-only targets in both wild type and Foxa1 mutant livers (Figure 5C, left panel). While none of Foxa2-only sites tested were bound by p53 in wild type livers, p53 was enriched at nine Foxa1-only sites, including the cell-cycle associated genes *Aatf* and *Zwint*, in wild type livers, and at two additional sites in Foxa1-deficient livers. In addition, expression of Zwint is significantly downregulated in livers of Foxa1 mutant mice, indicating that Foxa1 is essential for full activation of this gene and potentially regulates mRNA levels of Zwint in concert with p53 (Figure 5D).

For most sites examined, there was a trend toward increased enrichment of p53 binding in Foxa1-deficient livers. Foxa1 and p53 have been shown to have an antagonistic relationship at the distal promoter element of *Afp* gene, where the two transcription factors regulate expression of *Afp* in opposite directions, and Foxa1 binding is enhanced in p53-null livers [24]. Our data suggest that the inverse regulatory relationship between Foxa1 and p53 is a more general phenomenon.

Functional categories over-represented in the genes bound by only Foxa1 are associated with cell cycle regulation, which is not surprising as many of them are p53 targets (Figure 5E). ‘Cyclin-dependent kinase activity’ and ‘cancer’ are among the top biological pathway enriched among the Foxa1-only gene targets. Furthermore, numerous motifs that are over-represented in Foxa1-bound regions are associated with transcription factors that play an important role in cell cycle regulation, such as Hic1, Klf12 (repressor of Tfap2a), Tfap2a, and Smads (Table S3). Foxa1 has been shown to facilitate chromatin access to Smad transcription factors [25], mediators of Tgf-β signaling, a pathway implicated in cancer [26] and also enriched in Foxa1-only elements.

Our analysis also confirms a number of motifs found previously in regions bound by Foxa1, such as those of the androgen receptor (AR) and the upstream stimulatory factor (Usf) [27], [28]. Usf has been shown to interact with Srebp1 [29], and we also find that the Srebp1 motif is enriched in Foxa1-bound regions. In addition, consensus sequences for several transcription factors expressed in the kidney as well as the liver, are enriched in regulatory elements bound only by Foxa1. It is interesting to note that the proximal promoter of Foxa1 contains a motif for a kidney-enriched nuclear factor [30]. Hence, Foxa1, but not Foxa2, is expressed in the kidney [31] and, when deleted, causes nephrogenic diabetes insipidus [32]. Our analysis suggests that combination of regulatory motifs present in one tissue can also occur in a different tissue, preserving the relationship between the transcription factors that occupy these elements.

Foxa1 also binds to the *cis*-regulatory elements of additional transcription factors, their co-activators and targets genes, in regulatory feed-forward loops. Foxa1 has been shown to cooperate with the estrogen receptor (ER) in gene activation in breast cancer cell lines [33], [34]. Here we demonstrate that Foxa1 also binds to the regulatory regions of the gene encoding histone methyltransferase Smyd3, a coactivator for ER-mediated transcription. Thus, Foxa1 and its targets constitute a regulatory feed-forward loop, which likely contributes to Foxa1 function in ER-dependent cancers (Figure 5F). Foxa1_only sites are more evenly distributed near TSS (+/−100 Kb) than dual targets with a large proportion of sites within 10 Kb (Figure 5G).

### Foxa2-only sites have a moderately strong forkhead consensus and are associated with lipid metabolism genes

**Figure 6:**
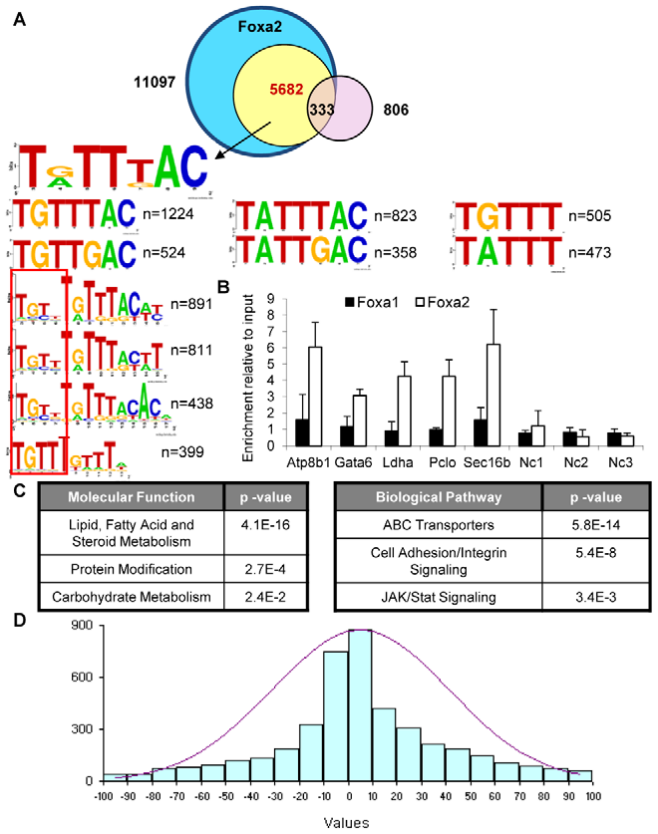
Foxa2-Specific Targets Contain a Medium-Strength *Forkhead* Consensus and Control Genes in Steroid and Lipid Metabolism. (A) The motifs found by *de novo* analysis for both Foxa2-only bound set (yellow circle) resemble a *forkhead* binding site. Intersection of Foxa2-bound regions and genes differentially expressed in livers of Foxa2-deficient mice (light purple circle) identified three-hundred thirty-three direct targets. (B) Validation of Foxa2-only targets by qPCR. Filled bars, ChIP of liver chromatin with an anti-Foxa1 antibody, open bars, ChIP with an anti-Foxa2 antibody. Binding is expressed as enrichment of the PCR amplicon relative to input DNA in liver chromatin. Negative controls (Nc1, Nc2, Nc3) are regions with a low amount of reads. (C) Foxa2 targets are enriched in genes involved in lipid and steroid metabolism, protein modification, and carbohydrate metabolism. (D) Histogram of the distribution of Foxa2-only sites relative to TSS. Foxa2-only sites, similar to the Foxa1/Foxa2 dual targets (compare Figure 4E), are distributed normally near transcription start sites (TSS), with most sites within ten kilobases (Kb) from TSS.

Motifs found by *de novo* analysis for both Foxa2-only set resemble a stronger *forkhead* binding site (Figure 6A) than for Foxa1-only regions. In fact, more than half of these sequences contain a perfect Foxa consensus (T[A/G]TT[G/T]AC). The top motifs from scanning analysis of the sequences with Transfac PWMs include numerous *forkhead* motifs (Table S1). In summary, both *de novo* and PWM scan analysis indicate that the sites bound by both Foxa factors contain the strongest match to the Foxa consensus, Foxa2-only regions the next strongest, and Foxa1-only sites have the weakest *forkhead* motif.

Surprisingly, in addition to the *forkhead* consensus, we found that sequences bound only by Foxa2 have a preference for the sequence “TGTT” immediately preceding the *forkhead* motif. Since the dual-bound regions demonstrate a preference for different flanking sequences, these data suggest that in addition to the strength of the *forkhead* motif, sequences surrounding the consensus also play a role in differential recruitment of Foxa factors to DNA.

Integrating the sites bound only bound by Foxa2 (Figure 6A, yellow circle) and gene expression changes in livers of Foxa2-deficient mice (Figure 6A, light purple circle) shows that a substantial fraction of these genes (three-hundred thirty-three or 41 percent) are direct targets of Foxa2. We also verified that several ChIP targets were indeed bound only by Foxa2 by qPCR (Figure 6B). Functional analysis of sites bound only by Foxa2 (Figure 6C) is consistent with previous reports that Foxa2 targets are enriched in genes involved in lipid and steroid metabolism [6] and carbohydrate metabolism [35]. ‘Abc transporters’, known targets of Foxa2 [6], and ‘JAK/Stat signaling’, are among the top biological pathway enriched in these targets. The motifs of other transcription factors found to be over-represented in the Foxa2-only bound sequences include liver-enriched Hnf1a and Hnf4a, also found in dual-bound regions, and homeodomain DNA binding proteins such as Nfil3, and Hmga1. Nfil3 has been shown to regulate Cyp7a1, the rate-limiting enzyme in bile acid synthesis [36] and likely controls expression of other genes crucial to bile acid homeostasis together with Foxa2. Foxa2-only sites, similar to dual-bound regions, are distributed near transcription start sites (TSS), with most sites within ten kilobases (Kb) from TSS (Figure 6D).

In summary, the closely-related transcriptional regulators Foxa1 and Foxa2 cooperate during development, and but are functionally diversified in the adult liver, as indicated by their target preferences *in vivo*.

## Discussion

Functional diversification of paralogous transcription factors can arise either by mutation in *cis*-regulatory elements or changes in the coding sequence of the proteins [2]. We examined the acquisition of novel biochemical functions by Foxa1 and Foxa2, the closest paralogs in the Foxa subfamily of winged-helix transcription factors, in the adult liver. Genome-wide location analysis (ChIP-Seq) revealed that Foxa1 and Foxa2 have unique targets in addition to many common ones, indicative of diverged function, which is also reflected in the divergent effects on the liver transcriptome by ablation of either factor. These results are consistent with an early study by Lai and colleagues who found that Foxa1 and Foxa2 (Hnf-3α and Hnf-3β) have different affinities for the two binding sites in the promoter of the TTR gene *in vitro* [8]. Differences in DNA binding by the Foxa paralogs could be the result of a handful of divergent residues in the *forkhead* domain itself, and a few amino acid residues located outside the DNA binding domain that are targeted by post-translational modifications. Among seven amino acids in the winged-helix DNA binding domain that differ between Foxa1 and Foxa2, five are conserved between Foxa1 and the remaining Foxa family member Foxa3 (Figure S1), indicating that Foxa1 and Foxa3 represent the ancient precursor gene, while Foxa2 has acquired new mutations at those positions.

Recently, Kohler and Cirillo have reported that acetylation of Foxa1 by p300 attenuates binding of Foxa1 to DNA [37]. Multiple putative acetylation sites, identified by *in silico* analysis, are divergent between Foxa1 and Foxa2 and likely contribute to their specific DNA binding properties. Additionally, a recent study implicated Foxa2 as a substrate for DNA-dependent protein kinase (DNA-PK), which targets serine 283 [13]. A mutation of that residue to alanine resulted in a protein with greater affinity for sequence-specific DNA-binding. Interestingly, Foxa1 has an alanine rather than a serine at position 283, evidence for another functional diversification of the two proteins. Cirillo and colleagues showed that the C-terminal domain of Foxa1 also enhances DNA-binding of the protein to the albumin enhancer [38]. While Foxa1 and Foxa2 share strong sequence conservation in their transactivation domains within the C-terminus, the remainder of the C-terminal domain is quite divergent between the two paralogs.

Binding of Foxa2 to its targets in the adult liver has been studied previously [15], [39], [40], [41], [42]. We reported that Foxa2 is required for normal bile acid homeostasis and a cluster of categories with genes involved in lipid and steroid metabolism was identified as bound by Foxa2 *in vivo* [6]. The previously reported data sets [4], [6] were comprised of all sites, including those bound by Foxa1 as well. We also showed that deletion of Foxa2 in hepatocytes affects expression of hundreds of genes in mice fed a standard diet and thousands of genes in mice on a cholic acid-enriched diet, demonstrating that Foxa1 cannot compensate for the loss of its paralog [4]. Here, we found that Foxa2-only sites are also associated with genes important to lipid metabolism and contain a medium-strength *forkhead* consensus, as well as motifs for liver-enriched transcription factors and nuclear receptors, and AT-rich motifs, including homeodomain transcription factors, Nfil3, and Hmga1. Hence, specialization of Foxa paralogs in the adult liver has resulted in Foxa2 acquiring a specific role in coordinating the transcriptional regulatory network that controls bile acid and lipid metabolism.

The function of Foxa1 has been studied primarily in a variety of cancer cell lines [34], [43] and, together with Foxa2, during embryonic development [14], [44], [45]. Binding of Foxa1 to its targets was shown to be required for chromatin-association of androgen receptor (AR) in prostate cancer cells [27], and the estrogen receptor (ER) and retinoic acid receptor (RAR), in breast cancer cell lines [34], [46]. Foxa1 was also implicated in cell cycle regulation in tumor-derived cells [33], [47], but a mechanistic model was not established. We found that Foxa1-only sites are enriched for binding sites of p53, a tumor suppressor that activates target genes that induce cell cycle arrest, apoptosis, or senescence. A single such composite Foxa1/p53 site was previously characterized in the promoter of the alpha-fetoprotein (*Afp*) gene [48], which is expressed during development and repressed in the adult hepatocyte. We found that a p53 motif is prevalent in numerous Foxa1-only targets in the adult liver and validated that p53 binds these sequences. In addition, we detected other motifs corresponding to factors that interact with p53 (Klf12 (repressor of Tfap2a), Tfap2a, and Smads) or compete with p53 for binding (Hic1) [49]. This is a novel function of Foxa1, which has implications for the role of Foxa1 in cell cycle progression and cancer.

A study by Gao and Matusik (personal communication) to detect potential DNA binding complexes occupying the TS2 regulatory element of the probasin gene, bound by Foxa1 in the prostate [27], identified poly (ADP-ribose) polymerase (Parp1) as a protein also interacting with this region. Parp1, a chromatin-associated enzyme, is involved in regulation of numerous processes, including proliferation, recovery from DNA damage, and tumor transformation. Parp1 modulates stability of p53 in unstressed cells [50] and interacts with androgen receptor (AR) [51], retinoic acid receptor (RAR) [52], proteins functionally linked with Foxa1, and may interact with Foxa1 itself. These data support a regulatory network unique to the Foxa1 paralog, one that functions in cellular growth and genome stability.

In summary, the transcriptional regulators Foxa1 and Foxa2 share a significant fraction of *cis*-regulatory elements that contain a high-affinity *forkhead* binding site and regulate genes essential in development and those implicated in etiology of diabetes. It is possible that gene regulatory networks of important disease susceptibility genes have redundant modules and closely related paralogs that compensate for each other, as occurs in developmental regulation. However, while Foxa1 retains the more ancient role of regulating proliferation and growth by influencing DNA binding of p53, Foxa2 has acquired mutations in its DNA binding domain and a new role in the hepatocyte, regulating genes involved in lipid metabolism. In this instance, it is more advantageous for duplicated paralogs to perform different functions and transduce the many different physiological signals propagated through differentiated tissues. We propose that this functional diversification of the Foxa paralogs contributed to the maintenance of both genes during evolution.

## Materials and Methods

### Animals

The derivation of *Foxa1* null and *Foxa1^loxP/loxP^* mice, the *Alfp.Cre* transgenic line to achieve hepatocyte-specific deletion of Cre, and the *Foxa2^loxP/loxP^;Alfp.Cre* mouse model has described previously [35], [44], [53]. Two- to three-months old male mice were used for all ChIP-Seq experiments. Embryos at 18.5 days of gestation were used for gene expression profiling. Mice were genotyped by PCR of tail DNA as described [35], [44], [53]. All animal experiments were conducted with approval of the Institutional Animal Care and Use Committee of the University of Pennsylvania.

### RNA isolation and expression analysis

Liver RNA was isolated from *Foxa1* null, *Foxa2^loxP/loxP^;Alfp.Cre*, *Foxa1* ^−/−^;*Foxa2^loxP/loxP^*;*Alfp.Cre* and control embryos (e18.5) and *Foxa1^loxP/loxP^;Alfp.Cre* and control mice (2–3 months), and quantitative reverse transcription-PCR performed as described [35]. Hybridization to Agilent 4×44k Whole Mouse Genome Oligo Microarray and microarray analysis were completed as reported previously [4]. Five individual samples for each genotype were analyzed for both embryonic and adult study.

### Chromatin immunoprecipitation and ChIP–Seq

ChIP and the following real time PCR reactions were performed as described [6]. Snap-frozen mouse liver (100 mg) from wild type mice was used to prepare chromatin. Foxa1–specific antiserum (a kind gift of G. Schütz, Heidelberg, Germany), Foxa2-speficic antiserum (a kind gift of J.A. Whitsett), and p53 antibody (Ab1 OP03, Calbiochem) were used for immunoprecipitation. ChIP-Seq was performed as reported previously [44].

### Western blot analysis

Protein analysis by immunoblot was performed as reported previously [6]. The primary antibodies used were guinea pig antibody to Foxa1 (1∶1000), rabbit antibody to Foxa2 (1∶5000) (both a kind gift of J.A. Whitsett) and rabbit antibody to TBP (1∶100, Santa Cruz, sc-273).

### Sequence analysis

Foxa1 ChIP libraries for five biological replicates and two input libraries were sequenced in a total of seven lanes to 36 nucleotides. The reads were aligned to the mouse genome (mm8; NCBI Build 36) using the ELAND aligner (Illumina). Reads with a unique best alignment were retained for further processing. We have pooled reads from previously published Foxa2 ChIP-Seq (4 biological replicates, sequenced on GAI) and 2 biological replicates sequenced on GAII, resulting in 28,625,810 total reads for Foxa1 ChIP and 32,852,268 total reads for Foxa2 ChIP. The GLITR algorithm was run 10 times on a random sample of 28 million reads each from Foxa1 or Foxa2 ChIPseq and input samples which were compared to a large pool of input reads from multiple mouse tissues. Regions were defined as bound and subjected to further analysis if identified by GLITR in 3 or more runs.

The set of all Foxa1 or Foxa2 sites was created by taking the union of regions from each GLITR analysis and merging regions that overlapped. The set of common sites was defined as the set of merged regions. To identify truly unique binding sites for each factor for subsequent analyses, we defined stringent criteria for Foxa1-only and Foxa2-only binding sites. The set of unique targets contains peaks for the first factor that include at most one tag per million per kilobase (Kb) for the other. All region manipulations were performed using Perl and R scripts in the TESS Location Analysis package.

CisFinder [54], RSAT [55], and SCOPE [56] software were used for *de novo* motif finding. Sequence alignment of Foxa proteins was performed by ClustalW2 algorithm [57]. Analysis of overrepresented functional categories was carried out as described previously [4].

The Asap software was used for positional weight matrix enrichment analysis (parameters: Fisher's exact test, sequence-based statistics, PWM Threshold 0.8) [58], scoring the matrices from Jaspar database and all vertebrate PWMs from TRANSFAC 2009.2 against positive and negative sequence sets. For each conservative (Foxa1_zero, Foxa2_zero) and semi-conservative set (Foxa1_one, Foxa2_one) set, analysis was conducted against two negative data sets: a corresponding set bound by the other factor (Foxa1_zero as positive, Foxa2_zero as negative) and a set of background genomic sequences not bound by either Foxa1 or Foxa2 (Foxa1_zero as positive, background as negative). The set of sequences bound by both Foxa1 and Foxa2 was scored against one negative background data set.

The microarray data from this study can be accessed at ArrayExpress (http://www.ebi.ac.uk/arrayexpress/) under accession nos. E-MEXP-2106, E-MEXP-3426, and E-MEXP-3428. The ChIP-seq data from this study can be accessed at GEO (http://www.ncbi.nlm.nih.gov/geo/) under accession nos. GSE25836, GSE26729, and GSE33666.

## Supporting Information

Figure S1 Alignment of All Members of Foxa Subfamily. Sequence alignment of mouse Foxa1 and Foxa2 proteins by ClustalW2 algorithm. The winged-helix DNA binding domain is highlighted in blue. Residues in the DNA-binding domain that are conserved between Foxa1 and Foxa3 are highlighted in yellow. ‘*’ (identical residues in all sequences), ‘:’ (highly conserved column), ‘.’ (weakly conserved column).
(PDF)

Table S1 Overrepresented Forkhead Positional Weight Matrices in Foxa1-specific, Foxa2-specific, and Dual ChIP-Seq Targets.
(XLS)

Table S2 Cis-regulatory Elements of Diabetes Susceptibility Genes Bound by Both Foxa1 and Foxa2.
(DOC)

Table S3 Overrepresented Other (non-Forkhead) Positional Weight Matrices in Foxa1-specific, Foxa2-specific, and Dual ChIP-Seq Targets.
(XLS)
