# Supplementary material for: Genome-Wide Location Analysis Reveals Distinct Transcriptional Circuitry by Paralogous Regulators Foxa1 and Foxa2
Source: PLoS Genet. 2012 Jun 21;8(6):e1002770. doi: 10.1371/journal.pgen.1002770 (PMC3380847; doi:10.1371/journal.pgen.1002770)
Supplement: Figure S1 — Alignment of All Members of Foxa Subfamily. Sequence alignment of mouse Foxa1 and Foxa2 proteins by ClustalW2 algorithm. The winged-helix DNA binding domain is highlighted in blue. Residues in the DNA-binding domain that are conserved between Foxa1 and Foxa3 are highlighted in yellow. ‘*’ (identical residues in all sequences), ‘:’ (highly conserved column), ‘.’ (weakly conserved column). (PDF) [file pgen.1002770.s001.pdf]

Bochkis et al., Supplemental Figure 1

```
Foxa1        MLGTVKMEGHESNDWNSYYADTQEAYSSVPVSNMNSGLGSMNSMNTYMTMNTMTT---SG 57
Foxa2        MLGAVKMEGLEPSDWSSYYAE-PEGYSSV--SNMNAGLG-MNGMNTYMSMSAAAMGGGSG 56
Foxa3        MLGSVKMEAHDLAEWS-YYPEAGEVYSPVN------PVPTMAPLNSYMTLNPLSSPYPPG 53
             ***:****. :  :*. **.:  * **.*        :  *  :*:**::.. :    .*

NM_008259    NMTPASFNMSYANTGLGAGLSPGAVAGMP---GASAGAMNSMTAAGVTAMGTALSPGGMG 114
NM_010446    NMSAGSMNMS---SYVGAGMSP-SLAGMSPGAGAMAGMSGSAGAAGVAGMGPHLSP-SLS 111
NM_008260    GLQASPLPTG--------PLAP--------------------PAPTAPLGPTFPS--LG 82
             .: ...:  .         ::*                     .* .: :*. :..  :.

NM_008259    SMGAQPATSMNGLGPYAAAMNPCMSPMAYAPSNLGRSRAGGGGDAKTFKRSYPHAKPPYS 174
NM_010446    PLGGQAAGAMGGLAPYAN-MN-SMSPM-YGQAGLSRAR-----DPKTYRRSYTHAKPPYS 163
NM_008260    TGGSTGGSASGYVAPGPG--------LVHGKE-----------MAKGYRRPLAHAKPPYS 123
             . *.  . : . :.* .         : :.              .* ::*. .*******

NM_008259    YISLITMAIQQAPSKMLTLSEIYQWIMDLFPYYRQNQQRWQNSIRHSLSFNDCFVKVARS 234
NM_010446    YISLITMAIQQSPNKMLTLSEIYQWIMDLFPFYRQNQQRWQNSIRHSLSFNDCFLKVPRS 223
NM_008260    YISLITMAIQQAPGKMLTLSEIYQWIMDLFPYYRENQQRWQNSIRHSLSFNDCFVKVARS 183
             ***********:*.*****************:**:*******************:**.**

NM_008259    PDKPGKGSYWTLHPDSGNMFENGCYLRRQKRFKCEKQPGAGGGSGGGGSKGGPESRKDPS 294
NM_010446    PDKPGKGSFWTLHPDSGNMFENGCYLRRQKRFKCEKQLALKEAAG-AASSGGKK-----T 277
NM_008260    PDKPGKGSYWALHPSSGNMFENGCYLRRQKRFKLEEK-AKKGNSATSASRNGTA------ 236
             ********:*:***.****************** *:: .    :. ..* .*

NM_008259    GPGNPSAESPLHRGVHGKASQLEGAPAPGPAASPQTLDHSG--ATATGG-ASELKSPASS 351
NM_010446    APGSQASQAQLGEAAGSASETPAGTESPHSSASPCQEHKRGGLSELKGAPASALSPPEPA 337
NM_008260    --GSATSATTTAATAVTSPAQPQPTPSEPEAQS--------------GDDVGGLDCASPP 280
               *. :: :     .   .     : :   : *              *  .. *. . ..

NM_008259    SAPPISSGPGALASVPPSHPAHGLAPHESQLHLKGDPHYSFNHPFSINNLMSSSE----- 406
NM_010446    PSPGQQQQAAAHLLGPPHHP--GLPP---EAHLKPEHHYAFNHPFSINNLMSSEQQHHHS 392
NM_008260    SSTPYFSG----LELP--------------GELKLDAPYNFNHPFSINNLMSEQT----- 317
             .:.   .        *               .** :  * ************..

NM_008259    ----QQHKLDFKAYEQALQYSP-YGATLPASLPLGSASVATRSPIEPSALEPAYYQGVYS 461
NM_010446    HHHHQPHKMDLKAYEQVMHYPGGYGSPMPGSLAMGPVTNKAGLDASPLAADTSYYQGVYS 452
NM_008260    -------------------------STPSKLDVG-----FGGYGAESGEPGVYYQSLYS 346
                                          . *..* :*             .    ***.:**

NM_008259    RPVLNTS 468
NM_010446    RPIMNSS 459
NM_008260    RSLLNAS 353
             *.::*:*
```
