# Supplementary material for: Genome-Wide Location Analysis Reveals Distinct Transcriptional Circuitry by Paralogous Regulators Foxa1 and Foxa2
Source: PLoS Genet. 2012 Jun 21;8(6):e1002770. doi: 10.1371/journal.pgen.1002770 (PMC3380847; doi:10.1371/journal.pgen.1002770)
Supplement: Table S2 — Cis-regulatory Elements of Diabetes Susceptibility Genes Bound by Both Foxa1 and Foxa2. (DOC) [file pgen.1002770.s003.doc]

**Supplemental Table 2**

| **Gene Symbol** | **Gene Name** | **Location (mm8)** | **Reference** |
| --- | --- | --- | --- |
| Arap1  (Centd2) | ArfGAP with RhoGAP domain, ankyrin repeat and PH domain 1 | chr7:101197192-101197405 |  |
| Ctla4 | cytotoxic T-lymphocyte-associated protein 4 | chr1:60804430-60804700 |  |
| Cp | ceruloplasmin (ferroxidase) | chr3:20157492-20157700 |  |
| Eif2ak3 | eukaryotic translation initiation factor 2 alpha kinase 3 | chr6:70743500-70743720 |  |
| Fto | fat mass and obesity associated | chr8:94269382-94269800 |  |
| Fto | fat mass and obesity associated | chr8:94293200-94293439 |  |
| Fto | fat mass and obesity associated | chr8:94385450-94385670 |  |
| Fto | fat mass and obesity associated | chr8:94419900-94420107 |  |
| Gatad2a | GATA zinc finger domain containing 2A | chr8:72896406-72896630 |  |
| Hex | hematopoietically expressed homeobox | chr19:37507613-37507914 |  |
| Hnf1a | HNF1 homeobox A | chr5:115231950-115232200 |  |
| Hnf1b | HNF1 homeobox B | chr11:83697700-83697970 |  |
| Hnf4a | hepatic nuclear factor 4, alpha | chr2:163232076-163232343 |  |
| Ifih1 | interferon induced with helicase C domain 1 | chr2:62453444-62453698 |  |
| Insr | insulin receptor | chr8:3256478-3256736 |  |
| Notch2 | Notch gene homolog 2 (Drosophila) | chr3:98130452-98130650 |  |
| Notch2 | Notch gene homolog 2 (Drosophila) | chr3:98130452-98130650 |  |
| Nr5a2 | nuclear receptor subfamily 5, group A, member 2 | chr1:138712420-138712700 |  |
| Nr5a2 | nuclear receptor subfamily 5, group A, member 2 | chr1:138746200-138746450 |  |
| Onecut1 | one cut homeobox 1 | chr9:74587700-74587950 |  |
| Pax4 | paired box gene 4 | chr6:28409269-28409479 |  |
| Pdx1 | pancreatic and duodenal homeobox 1 | chr5:147610000-147610255 |  |
| Pparg | peroxisome proliferator-activated receptor gamma | chr6:115384646-115384850 |  |
| Pparg | peroxisome proliferator-activated receptor gamma | chr6:115432300-115432548 |  |
| Rbms1 | RNA binding motif, single stranded interacting protein 1 | chr2:60603760-60603960 |  |
| Slc30a8 | solute carrier family 30 (zinc transporter), member 8 | chr15:51926173-51926350 |  |
| Sorcs1 | sortilin-related VPS10 domain containing receptor 1 | chr19:49429150-49429334 |  |
| Sorcs1 | sortilin-related VPS10 domain containing receptor 1 | chr19:50908265-50908490 |  |
| Sorcs3 | sortilin-related VPS10 domain containing receptor 3 | chr19:48708078-48708327 |  |
| Srebf1 | sterol regulatory element binding transcription factor 1 | chr11:60056781-60056990 |  |
| Thada | thyroid adenoma associated | chr17:84096800-84097035 |  |
| Thada | thyroid adenoma associated | chr17:84159849-84160085 |  |
| Zfand6 | zinc finger, AN1-type domain 6 | chr7:84562750-84562960 |  |
| Tcf7l2 | transcription factor 7-like 2 (T-cell specific, HMG-box) | chr19:55797560-55797770 |  |

**Supplemental References**

1. Saxena R, Elbers CC, Guo Y, Peter I, Gaunt TR, et al. (2012) Large-Scale Gene-Centric Meta-Analysis across 39 Studies Identifies Type 2 Diabetes Loci. Am J Hum Genet 90: 410-425.

2. Ueda H, Howson JM, Esposito L, Heward J, Snook H, et al. (2003) Association of the T-cell regulatory gene CTLA4 with susceptibility to autoimmune disease. Nature 423: 506-511.

3. Logan JI, Harveyson KB, Wisdom GB, Hughes AE, Archbold GP (1994) Hereditary caeruloplasmin deficiency, dementia and diabetes mellitus. Qjm 87: 663-670.

4. Senee V, Vattem KM, Delepine M, Rainbow LA, Haton C, et al. (2004) Wolcott-Rallison Syndrome: clinical, genetic, and functional study of EIF2AK3 mutations and suggestion of genetic heterogeneity. Diabetes 53: 1876-1883.

5. Consortium WTCC (2007) Genome-wide association study of 14,000 cases of seven common diseases and 3,000 shared controls. Nature 447: 661-678.

6. Sladek R, Rocheleau G, Rung J, Dina C, Shen L, et al. (2007) A genome-wide association study identifies novel risk loci for type 2 diabetes. Nature 445: 881-885.

7. Yamagata K, Oda N, Kaisaki PJ, Menzel S, Furuta H, et al. (1996) Mutations in the hepatocyte nuclear factor-1alpha gene in maturity-onset diabetes of the young (MODY3). Nature 384: 455-458.

8. Horikawa Y, Iwasaki N, Hara M, Furuta H, Hinokio Y, et al. (1997) Mutation in hepatocyte nuclear factor-1 beta gene (TCF2) associated with MODY. Nat Genet 17: 384-385.

9. Yamagata K, Furuta H, Oda N, Kaisaki PJ, Menzel S, et al. (1996) Mutations in the hepatocyte nuclear factor-4alpha gene in maturity-onset diabetes of the young (MODY1). Nature 384: 458-460.

10. Liu S, Wang H, Jin Y, Podolsky R, Reddy MV, et al. (2009) IFIH1 polymorphisms are significantly associated with type 1 diabetes and IFIH1 gene expression in peripheral blood mononuclear cells. Hum Mol Genet 18: 358-365.

11. Bruning JC, Winnay J, Bonner-Weir S, Taylor SI, Accili D, et al. (1997) Development of a novel polygenic model of NIDDM in mice heterozygous for IR and IRS-1 null alleles. Cell 88: 561-572.

12. Florez JC, Jablonski KA, Kahn SE, Franks PW, Dabelea D, et al. (2007) Type 2 diabetes-associated missense polymorphisms KCNJ11 E23K and ABCC8 A1369S influence progression to diabetes and response to interventions in the Diabetes Prevention Program. Diabetes 56: 531-536.

13. Gannon M, Ray MK, Van Zee K, Rausa F, Costa RH, et al. (2000) Persistent expression of HNF6 in islet endocrine cells causes disrupted islet architecture and loss of beta cell function. Development 127: 2883-2895.

14. Shimajiri Y, Sanke T, Furuta H, Hanabusa T, Nakagawa T, et al. (2001) A missense mutation of Pax4 gene (R121W) is associated with type 2 diabetes in Japanese. Diabetes 50: 2864-2869.

15. Ahlgren U, Jonsson J, Jonsson L, Simu K, Edlund H (1998) beta-cell-specific inactivation of the mouse Ipf1/Pdx1 gene results in loss of the beta-cell phenotype and maturity onset diabetes. Genes Dev 12: 1763-1768.

16. Altshuler D, Hirschhorn JN, Klannemark M, Lindgren CM, Vohl MC, et al. (2000) The common PPARgamma Pro12Ala polymorphism is associated with decreased risk of type 2 diabetes. Nat Genet 26: 76-80.

17. Grant SF, Thorleifsson G, Reynisdottir I, Benediktsson R, Manolescu A, et al. (2006) Variant of transcription factor 7-like 2 (TCF7L2) gene confers risk of type 2 diabetes. Nat Genet 38: 320-323.
